# Editorial: Cognitive, Affective, Behavioral, and Multidimensional Domain Research in STEM Education: Active Approaches and Methods Towards Sustainable Development Goals (SDGs)

**DOI:** 10.3389/fpsyg.2022.881153

**Published:** 2022-03-31

**Authors:** Jin Su Jeong, David González-Gómez

**Affiliations:** Departamento de Didáctica de las Ciencias Experimentales y Matemáticas, Universidad de Extremadura, Cáceres, Spain

**Keywords:** STEM education, active methodologies, sustainability, climate change, innovative, sustainable education, educational psychology, Sustainable Development Goals (SDGs)

This Research Topic consists of 15 articles on various aspects and methodologies of active and sustainable Science, Technology, Engineering, and Mathematics (STEM) education toward educational psychology domain research contributed to by 49 authors. At the time of writing the Research Topic, the articles published have been checked online more than 24,000 times and received abundant citations in the scientific literature.

The topic theme, “STEM education,” is an umbrella term for the approaches and methods from different research disciplines and areas, targeted at the improvement of Sustainable Development Goals (SDGs) and the cognitive, affective, behavioral, and multidimensional domain in both academic, professional, and industrial personnel who are involved in this field with their everyday work. Here, the various educational psychology domains are getting increased attention in relation to STEM and SDGs. In the same context, it is still challenging and inspiring because this educational psychology domain needs to reflect the current renewable and sustainable situations that are required to follow and achieve the SDGs in STEM education. With various active approaches and methods becoming available, all these ideas and thoughts in the educational psychology domain could be integrated into the area of STEM toward the SDGs that can diagnose and analyze various peoples' cognitive, affective, behavioral, and multidimensional patterns as well as their peculiarities.

Therefore, this Research Topic aims to offer and contribute to a corpus of solid research for concentrating and addressing the challenges required to stipulate an adequate STEM and Sustainable Development Education (SDE) toward SDGs to scholars and professionals of different educational degrees and backgrounds in the cognitive, affective, behavioral, and multidimensional research domain. Although the SDGs are progressively establishing a part of the curricula of numerous educational institutions, efforts must be made to ensure proper implementation and development of SDE, and interdisciplinary study of sustainability-oriented topics looking for the SDGs in higher education, as well as fresh viewpoints on current and existing challenges.

The 15 articles discuss the major trends in active and sustainable STEM education toward educational psychology domain research. The articles are now summarized in the order of articles most viewed in the topic proposed (the highest rank is “1”):

1. *A STEM Course Analysis During COVID-19: A Comparison Study in Performance and Affective Domain of PSTs Between F2F and F2S Flipped Classroom* (Jeong and González-Gómez). This is an original research article to compare and examine two different instruction situations with an identical teaching methodology, Face-To-Face (F2F) and Face-To-Screen (F2S) flipped methodology, on the performance and affective domain of Pre-Service Teachers (PSTs) in a STEM course. Here, the results indicated F2F was preferred over F2S although F2F-F2S transition was an effective procedure. Thus, it will allow PSTs to be more interactive in an online setting for their future application iof STEM courses (5267 total views).
2. *Student Engagement in Mathematics Flipped Classrooms: Implications of Journal Publications From 2011 to 2020* (Lo and Hew). This is a systematic review to examine the comparative studies' results, which were published between 2011 and 2020. It summarized this instructional approach effects along with traditional lecturing on the behavioral, emotional, and cognitive engagement of students within mathematics courses. Here, the results had significant implications for future flipped-classroom practice such as students solving real-life problems, and for research on student engagement in mathematics education (2,751 total views).
3. *An Exploratory Study Interrelating Emotion, Self-Efficacy, and Multiple Intelligence of Prospective Science Teachers* (Hernández-Barco et al.). This is an original research article to identify the learning styles of science teachers along with the theory of multiple intelligences. It studies their self-efficacy perception concerning the different scientific contents they would be required to instruct in and ascertaining correlations between these variables. Here, the results showed that these future teachers received greater refusal toward Physics and Chemistry than toward Biology and Geology. Thus, it is conceivable to establish emotional differences that the future teachers felt toward science according to which track they took in their pre-university backgrounds (2,102 total views).
4. *Promoting Middle School Students' Science Text Comprehension via Two Self-Generated “Linking” Questioning Methods* (Sason et al.). This is an original research article to measure two forms of reading approaches as a quasi-experimental study: Self-generated questions either connecting to prior knowledge (Extra-Text) or connecting between the text's parts (Within-Text). Here, the results from both short- and long-term evaluations indicated that those learners trained to generate questions about within-text connections reached significantly higher science text comprehension achievements than the other groups. Also, the findings may contribute to the support methods' design and teaching approaches for supporting general literacy and, in particular, scientific literacy (2,014 total views).
5. *When to Scaffold Motivational Self-Regulation Strategies for High School Students' Science Text Comprehension* (Michalsky). This is an original research article to examine the important role motivation plays in reading comprehension for science students in a 14-week quasi-experimental study. Here, outcomes suggested meta-motivational scaffolding delivery as a possibly important means for reassuring the scientific literacy of students and effortful determination with challenging science assignments. Especially, it was at the reflection-before-action phase for looking ahead and at the reflection-on-action stage for looking back. However, for this preliminary study, more theoretical and practical implications were deliberated to address the increasing challenges in science schoolwork (1774 total views).
6. *A Study of Disposition, Engagement, Efficacy, and Vitality of Teachers in Designing Science, Technology, Engineering, and Mathematics Education* (Lin et al.). This is an original research article to propose and test a theoretical model of how STEM e-learning affects teachers' perceptions of engagement, disposition, and efficacy, which can affect their vitality when they design STEM education. Here, the teachers' disposition could predict lesson design engagement. For both factors, they predicted effectiveness for designing STEM e-learning in turn, showing that well suited STEM instructors must not only be able to plan a STEM curriculum but also have a positive STEM education perception (1,218 total views).
7. *Emotional and Cognitive Preservice Science Teachers' Engagement While Living a Model-Based Inquiry Science Technology Engineering Mathematics Sequence About Acid-Base* (López-Banet et al.). This is a brief research report article focusing on the requirements of science teachers to carefully check the classroom teaching methodologies, to confirm that students are given chances to improve proper comprehension of acid/base models and concepts. The results indicate that there are noteworthy relationships between emotions and knowledge, which are different according to the skill concerned. Also, substantial correlations between emotions have been discovered (1,053 total views).
8. *Evolution of Prospective Secondary Education Economics Teachers' Personal and Emotional Metaphors* (Mellado et al.). This is an original research article to examine prospective economics teachers' personal and emotional metaphors about the roles as teachers. Then, their students examined their drawings and answers to open questions. The results showed that in the role of teachers, the most common metaphors used in both questionnaires were cognitivist and constructivist. Also, the findings' comparison before and after the teaching practicum exposed that there were no changes in most of the participants' metaphors and linked models (1,023 total views).
9. *Comparison Between Performance Levels for Mathematical Competence: Results for the Sex Variable* (García Perales and Palomares Ruiz). This is an original research article to utilize an ex post facto. It is a descriptive and quantitative methodology to assess the 3,795 5th-year elementary school students' results, using the online version of the Evaluation Battery for Mathematical Ability (BECOMA On). Here, the results were also examined based on sex that showed statistically significant differences in the highest performance level. Thus, this research emphasized a diagnostic breach in the higher capacity students' identification, showing education systems' pending challenge for the educational inclusion of all students (878 total views).
10. *Detailed Emotional Profile of Secondary Education Students Toward Learning Physics and Chemistry* (Dávila-Acedo et al.). This is an original research article to present research needs to classify the K-7 to K-10 students' emotions toward Physics and Chemistry learning. Currently, there is a decreasing number of students who select programs associated with science. The results indicated that a decrease was noticed in the positive emotions' mean rate, which are joy, fun, and tranquility from K-8 to K-10. An increase in negative emotions, such as boredom, anxiety, disgust, fear, nervousness, worry, and sadness, was also detected (790 total views).
11. *Endorsing Sustainable Enterprises Among Promising Entrepreneurs: A Comparative Study of Factor-Driven Economy and Efficiency-Driven Economy* (Raza Sargani et al.). This is an original research article on the Theory of Planned Behavior (TPB), which aimed to scrutinize the relationship between predecessors on sustainable enterprise purpose and sustainable value formation. The research features the work values' importance in choosing programs for sustainability-oriented entrepreneurship. It can help candidates to advance their entrepreneurial capabilities and knowledge platform, which will inspire them to become sustainable upcoming entrepreneurs (753 total views).
12. *Prompting Socially Shared Regulation of Learning and Creativity in Solving STEM Problems* (Michalsky and Cohen). This is an original research article to check the influence of three support types (question prompts intended to support Socially Shared Regulation of Learning (SSRL), creative thinking, or a combination of both) on the individual participations in SSRL procedures and on their knowledge attainment. It used a sample of 104 seventh graders in accelerated science classes. Here, the findings fortify the SSRL-directed question prompts' case as a means to improve students' engagement in problem-solving jobs (661 total views).
13. *Effectiveness of Metacognitive Regulation Intervention on Attention-Deficit–Hyperactivity Disorder Students' Scientific Ability and Motivation* (Zheng et al.). This is an original research article to investigate the Metacognitive Regulation (McR) intervention effect on Attention-Deficit–Hyperactivity Disorder (ADHD) students' astronomy knowledge attainment and learning motivation. After a 15 week intervention, the results displayed that the experimental group of students achieved significantly better than the control group ones in learning motivation, scientific abilities, and metacognition. Also, the findings recommended that the McR intervention is an effective method for enlightening the ADHD students' learning abilities for science knowledge (532 total views).
14. *A Preliminary Study Comparing Pre-Service and In-Service School Principals' Self-Perception of Distributed Leadership Competencies in relation to Teaching and Managerial Experience* (Cebrián et al.). This is an original research article to validate the works that have concentrated on studying the self-conception of school principals. It showed their dispersed leadership competencies regarding their teaching and managerial experience. This preliminary work offers visions into the relevance of pre-service or in-service school principals with training and professional development programs on sustainability. It disseminated leadership that allows them to authentically retain the school community, advance innovative instructions, and lead change toward more sustainable schools (26 total views).
15. *The Mediating Role of Critical Thinking in the Relationship between EFL Learners' Writing Performance and their Language Learning Strategies* (Esmaeil Nejad et al.). This is an original research article to investigate the intervention ability of Critical Thinking (CT) between the important means of language learning and English as a Foreign Language (EFL) learners' writing approaches. Here, the results presented that there was a substantial relationship among (a) learning strategies and learners' writing performances, (b) the sub-sets of learning strategies and learners' writing performances, and (c) CT and learners' learning strategies. Thus, the findings may offer a view into facilitating EFL learners to contemplate and write more critically (15 total views).

STEM education can be contemplated as inter- and multi-disciplinary interactions for forming fundamental factors in teaching and learning (Jeong and González-Gómez; Lin et al.; Michalsky and Cohen). Here, for sustainable development and SDGs, STEM education can be associated with knowledge-acting values of sustainability education (Raza Sargani et al.; Cebrián et al.). Also, there is a robust connection to science and mathematics education in the same context of sustainable development and SDGs (Sason et al.; Lo and Hew; García Perales and Palomares Ruiz). However, it is still not associated with a distinct research area that could have its own values, approaches, dimensions, aptitudes, and scientific skills. Equally, in various educational levels, sustainability STEM was a starting step although they had implemented different transforming societies/cultures by hiring academics, leaders, and entrepreneurs as key personnel (Hernández-Barco et al.; Michalsky; Mellado et al.; Dávila-Acedo et al.; López et al.; Jeong and González-Gómez). Accordingly, it was crucial to reflect elementary-, mid-, high-schools' (García Perales and Palomares Ruiz; Michalsky and Cohen; Hernández-Barco et al.; Michalsky; Mellado et al.; Dávila-Acedo et al.), universities' (López et al.; Jeong and González-Gómez; Zheng et al.; Lo and Hew), and professionals' (Raza Sargani et al.; Esmaeil Nejad et al.) characteristics that were shifting moderately and slowly.

The decade of education for sustainability development (DESD) of the United Nations Educational, Scientific, and Cultural Organization (UNESCO) in the UN and UNESCO 2015–2030 Agenda amalgamated the beliefs, philosophies, purposes, and movements of sustainable education and SDE (Jeong and González-Gómez). In the context of challenging circumstances mentioned earlier, a pedagogical possibility can link current educational structures' niche with long-term and lifelong sustainability STEM education (Raza Sargani et al.; Cebrián et al.; Jeong and González-Gómez). In the various educational domains, it should be a segment of a universal procedure offering sustainability STEM education (Lin et al.; Michalsky and Cohen). Also, it can outline its aims together with information and knowledge to individuals that will readdress the effects of their aspect (García Perales and Palomares Ruiz; Esmaeil Nejad et al.). Particularly, it was enhanced to offer better comprehension of notions regarding STEM sustainability, was designed for the realization of information, knowledge, abilities, and worth, and was reorientated to the curricula in sustainability STEM education (Jeong and González-Gómez; Lin et al.; Michalsky and Cohen). Here, sustainability education can be directed into transformative learning, which was a cultural modification in STEM education for possible awareness and social, economic, and environmental interdependence for individuals. Thus, in the same transformative learning environment, it highlighed the importance of instructors who could support students who should realize much more self-regulating and consistent objectives (Michalsky; Mellado et al.; Zheng et al.).

In the context of educational psychology, much of the research indicated a cognitive, affective, behavioral, and multidimensional domain in both academic and industrial people who are involved in this field in their daily work (Mellado et al.; Hernández-Barco et al.; Zheng et al.; Jeong and González-Gómez). Here, different types of positive emotions fostered and emphasized motivation, metaphor, interest, and competence. Students were experienced in the aspect of the proposed questions and its practicality and were encouraged by a collaborative and cooperative environment (Hernández-Barco et al.; Michalsky; Mellado et al.; López-Banet et al.; García Perales and Palomares Ruiz; Dávila-Acedo et al.). Active approaches and methods also had a positive contribution to the educational psychology domain, with students' achievement, motivation, metaphor, competence, and collaboration improving in their class (Jeong and González-Gómez; Lo and Hew; López-Banet et al.; Michalsky). Along with the context mentioned in various teaching and learning methods, emotions played a primary position because they were highly associated with the cognitive feature. Positive emotions' presence for students in a subject aided their learning whereas negative emotions' generation restricted it (Hernández-Barco et al.; Michalsky; Mellado et al.; López-Banet et al.; García Perales and Palomares Ruiz; Dávila-Acedo et al.). For various students and professionals at different levels, it indicated that considering the negative familiarities and problems with sustainability STEM education could allow educators to better comprehend their negative attitude and low self-efficacy toward sustainability STEM teaching. It extended to the interrelation between self-efficacy, beliefs, and attitudes toward sustainability STEM if changes could lead to their teaching practices' improvement (López-Banet et al.; Jeong and González-Gómez; Hernández-Barco et al.; Mellado et al.). Therefore, it is essential to address specific research on cognitive, affective, behavioral, and multidimensional domain research in STEM education along with active approaches and methods toward SDGs.

The Research Topic offered a substantial number of articles on STEM education issues related to sustainability and educational psychology (Jeong and González-Gómez; Lin et al.; Michalsky and Cohen), incorporating SDGs in various levels (Raza Sargani et al.; Cebrián et al.), active methods (like flipped classroom) to improve engagement in mathematics education (Lo and Hew), middle-school science literacy achievements as an educational psychology (Sason et al.), pre-service teachers' affective domain study toward science courses (Hernández-Barco et al.), high-school students' science motivation as an active scaffolding method (Michalsky), emotional metaphors for economic teachers in secondary education (Mellado et al.), emotional and cognitive aspect for pre-service science teacher (López-Banet et al.), mathematical competence and performance comparison (García Perales and Palomares Ruiz), secondary students' detailed emotional profiles toward physics and chemistry (Dávila-Acedo et al.), metacognitive regulation intervention for ADHD students' scientific ability and motivation (Zheng et al.), and the critical thinking mediating role for EFL learners' writing performance and strategies (Esmaeil Nejad et al.).

Therefore, this Research Topic demonstrated and reiterated the results of theoretical, methodological, and empirical research on teaching and learning, competencies and assessment, policy, program development and implementation, instructor preparation, community- and project-based learning, institutional collaborations and partnerships, and other relevant subjects. With 15 articles published, we can observe that special emphasis was placed upon innovative teaching approaches and methodologies. They have been proven to be relevant on STEM education, not only considering the cognitive domain of the learning process but also the affective, behavioral, and multidimensional domains that all improved and were targeted toward furthering the SDGs.

## Author Contributions

All authors listed have made a substantial, direct, and intellectual contribution to the work and approved it for publication.

## Conflict of Interest

The authors declare that the research was conducted in the absence of any commercial or financial relationships that could be construed as a potential conflict of interest.

## Publisher's Note

All claims expressed in this article are solely those of the authors and do not necessarily represent those of their affiliated organizations, or those of the publisher, the editors and the reviewers. Any product that may be evaluated in this article, or claim that may be made by its manufacturer, is not guaranteed or endorsed by the publisher.

